# The Effect of Caffeic Acid on Zn Corrosion in NaCl: Electrochemical Studies

**DOI:** 10.3390/molecules30173648

**Published:** 2025-09-08

**Authors:** Aleksander Kucharek, Elżbieta Kuśmierek, Ewa Chrześcijańska, Waldemar Maniukiewicz, Jacek Rogowski, Aleksandra Bednarek, Andrzej Żarczyński

**Affiliations:** Institute of General and Ecological Chemistry, Lodz University of Technology, ul. Zeromskiego 116, 90-924 Lodz, Poland; ewa.chrzescijanska@p.lodz.pl (E.C.); andrzej.zarczynski@p.lodz.pl (A.Ż.)

**Keywords:** green corrosion inhibitor, caffeic acid, zinc corrosion, electrochemical studies, surface morphology

## Abstract

Caffeic acid (CA) can be applied as a green corrosion inhibitor for metals and alloys. The inhibition properties of caffeic acid for Zn in 0.1 M NaCl were investigated using electrochemical methods. The changes in Zn morphology were studied via scanning electron microscopy coupled with energy dispersive spectroscopy (SEM-EDS), X-ray diffraction (XRD) and time-of-flight secondary ion mass spectrometry (TOF-SIMS) techniques. Potentiodynamic polarisation (PDP) and electrochemical impedance spectroscopy (EIS) measurements proved that caffeic acid applied in the form of coatings on Zn surface was more effective than the addition of CA to NaCl. Furthermore, CA coatings revealed better corrosion protection with increasing duration of immersion. The highest inhibition efficiency was achieved for CA coating obtained from ethanol solution of CA (10 mM), and its value was almost 95%. The positive impact of CA coatings on the corrosion of Zn surface was confirmed with SEM-EDS, XRD and TOF-SIMS measurements. They proved not only the presence of CA on the Zn surface but also noticeably a lower amount of Zn corrosion products.

## 1. Introduction

Corrosion is a highly destructive process affecting various materials, whose cost is estimated as equivalent to about 3–4% of each nation’s gross domestic product (GDP). Application of available corrosion protection methods saves approximately 15 to 35% of the costs generated by corrosion [1]. Thus, it is crucial to prevent corrosion with proper methods which are not only effective but also environmentally friendly and cost-efficient. Corrosion affects various materials in a negative way, especially metals and alloys. Although zinc is regarded as a metal relatively resistant to corrosion, it does not mean that it is not affected by corrosion at all. Zn is widely used in various branches of industry. Considering its worldwide production and consumption, Zn ranks fourth among the metals [2,3]. Its main applications include coating and galvanising iron articles as well as use as an anode material in batteries [4]. Zinc is also applied in the automobile industry [5]. Moreover, zinc alloys are used in electrical components, industrial boilers and household articles [3]. Zn belongs to biodegradable metals and is an essential trace element in human physiology responsible for basic biological functions [6]. Considering its standard potential, Zn seems to undergo corrosion with an intermediate corrosion rate [7].

Corrosion protection of zinc and its alloys is also especially important with regard to historical manufactured goods, ancient and historical artworks, and their conservation [8,9]. Zinc is also, alongside copper, a component of brass, which is an alloy that has been widely used since the Bronze Age [10,11]. In the case of historical heritage artefacts, the effects of corrosion are undoubtedly more severe, since apart from the aforementioned costs, huge historical value is being irreversibly lost [12]. Therefore, it is necessary to develop effective and ecological methods of protecting such objects.

Corrosion inhibitors are commonly used in corrosion protection and are proving to be an excellent solution to the corrosion issues [13,14,15]. However, classic corrosion inhibitors, e.g., benzotriazoles, are often harmful to the environment and human health [16,17]. Thus, safe and ecological corrosion inhibitors (so-called green corrosion inhibitors) have recently drawn a lot of attention. Caffeic acid, being a derivative of cinnamic acid [18], can be an example of such a green inhibitor. This compound naturally occurs in many plants, e.g., coffee or tea [19], carrot, tomato, strawberry and blueberry [20,21]. Moreover, CA has health-beneficial properties resulting from its antioxidating and anti-inflammatory characteristics [22]. Green corrosion inhibitors, in general, have proven to be efficient in corrosion protection of a variety of metals and alloys such as zinc [23,24,25,26,27], copper [28,29,30,31,32], brass [11,33,34,35,36] and bronze [37,38,39,40,41]. A review of the literature reveals that there are not many papers describing the application of caffeic acid in corrosion protection. Thus far, this green inhibitor was successfully applied for copper [42], mild steel [43] and Mg alloys protection [44].

In this paper, the effect of caffeic acid on the corrosion of Zn was investigated in a NaCl solution. It was proved that chlorides have a significant effect on the corrosion rate of Zn [2]. Furthermore, NaCl accelerates the atmospheric corrosion of Zn, and that is why this medium was selected for our investigations. Caffeic acid seems to be a good corrosion inhibitor for historical heritage artefacts since it does not affect the colour and appearance of the protected materials. The effect of CA on Zn corrosion was investigated by modifying corrosive medium (NaCl) as well as by coating Zn surface with CA. Studies were performed with the application of electrochemical methods such as potentiodynamic polarisation (PDP) with OCP (open circuit potential) determination and electrochemical impedance spectroscopy (EIS). Additionally, scanning electron microscopy (SEM-EDS), X-ray diffraction (XRD) and time-of-flight secondary ion mass spectrometry (TOF-SIMS) techniques were applied to study the changes in morphology of Zn samples. The obtained results show that the application of CA as a corrosion inhibitor for historical heritage artefacts is quite promising.

## 2. Results and Discussion

### 2.1. Electrochemical Studies

PDP curves were recorded for pure Zn in 0.1 M NaCl in three consecutive experiments preceded by an OCP measurement. The same experiments were performed in 0.1 M NaCl modified by the addition of CA at various concentrations as well as for Zn with CA coatings. The PDP curves were transformed to Tafel plots to determine the corrosion parameters. A cathodic part of Tafel plots stands for the reduction of oxygen and hydrogen evolution at lower potentials (below −1.25 V in NaCl) according to the following reactions [2,45,46]:

O_2_ + 2H_2_O + 4e^−^ → 4OH^−^ (1)

2H_2_O + 2e^−^ → 2OH^−^ + H_2_ (2)

The anodic part of Tafel plots is attributed to metal dissolution followed by the formation of various corrosion products according to the following reactions:

Zn → Zn^2+^ + 2e^−^ (3)

Zn^2+^ + 2OH^−^ → Zn(OH)_2_ (4)

Zn(OH)_2_ → ZnO + H_2_O (5)

Zn(OH)_2_ + 2OH^−^ → Zn(OH)_4_^2−^ (6)

5Zn^2+^ + 8OH^−^ + 2Cl^−^ + H_2_O → Zn_5_(OH)_8_Cl_2_⋅H_2_O (7)

5Zn(OH)_2_ + 2Cl^−^ + H_2_O → Zn_5_(OH)_8_Cl_2_⋅H_2_O + 2OH^−^ (8)

In the first step, zinc dissolution results in the formation of a Zn(OH)_2_ and ZnO layer on the substrate. The layer of corrosion products can inhibit Zn corrosion. However, Zn(OH)_2_ further reacts with OH^−^ ions forming Zn(OH)_4_^2−^ ions (reaction 6) as well as with Cl^−^ ions forming zinc hydroxychloride (reaction 8). The products are deposited around pits [45].

#### 2.1.1. Corrosion Inhibition via CA Addition

**Figure 1:**
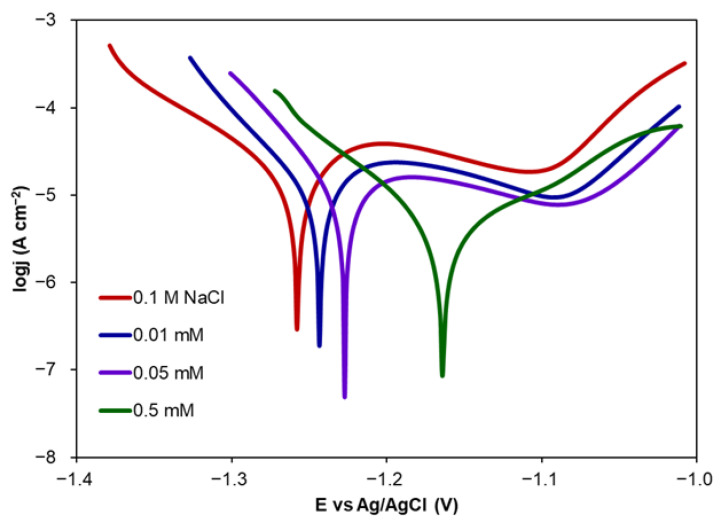
Exemplary Tafel curves recorded for Zn in 0.1 M NaCl modified by the addition of CA at different concentrations.

In order to check the inhibitory effect of CA against Zn corrosion in the presence of chloride ions, PDP curves were recorded in a NaCl solution modified by the addition of CA. Exemplary curves are shown in Figure 1.

The corrosion parameters such as corrosion potential (E_corr_), corrosion current density *(j_corr_*), corrosion rate (*CR*), anodic (b_a_) and cathodic (b_c_) Tafel slopes were determined from Tafel curves, and were used in calculating the corrosion rate (*CR*) and inhibition efficiency (*IE*). Their values are presented in Table 1.

The addition of CA at increasing concentrations resulted in an increase in E_corr_ and decrease in *j_corr_* values compared to the blank sample. The increase in E_corr_ values proves that the tested samples are less thermodynamically susceptible to corrosion processes. The decrease in *j_corr_* values results in lower *CR* values and proves the inhibitive effect of CA.

The highest *IE* value (82%) was observed for a CA concentration of 0.1 mM. *IE* values decreased for concentrations of CA higher than 0.1 mM. Thus, a CA concentration of 0.1 mM was found to be the most effective. The increase in *j_corr_* for a higher CA concentration (0.5 mM) can be explained by the fact that the thickness of the adsorption layer decreases. Taking into consideration that the capacity of the electrical double layer, determined via EIS measurements described below, increases for the concentration of 0.5 mM, it can be concluded that the thickness of the double layer decreases, and as an effect, the thickness of the adsorption layer also decreases [47]. The increasing number of inhibitor molecules near the Zn surface can result in the increasing number of intermolecular interactions and change how the CA molecules are adsorbed. The adsorption of the CA on Zn surface can be worsened by the higher number of repulsive interactions between CA molecules.

An increasing CA concentration resulted in an increase in OCP and E_corr_ values. However, this increase was not higher than 85 mV (except for CA concentrations higher than 0.1 mM) towards more positive values indicating that CA belongs to mixed-type inhibitors [48,49] with more control of the anodic reaction, i.e., metal dissolution [3]. The comparison of Tafel slopes (b_a_ and b_c_) shows that the addition of CA to NaCl causes only slight changes in their values. This means that the corrosion mechanism remains unchanged [48].

The lower *j_corr_* values are due to the adsorption of CA on Zn surface. The coverage of a metal surface by a corrosion inhibitor is closely related to the inhibition efficiency (Table 1). The degree of surface coverage (*Θ*) can be calculated using corrosion inhibition efficiency determined from PDP measurements according to the following equation [50]:

(9) $$\theta =\frac{IE}{100}$$

**Figure 2:**
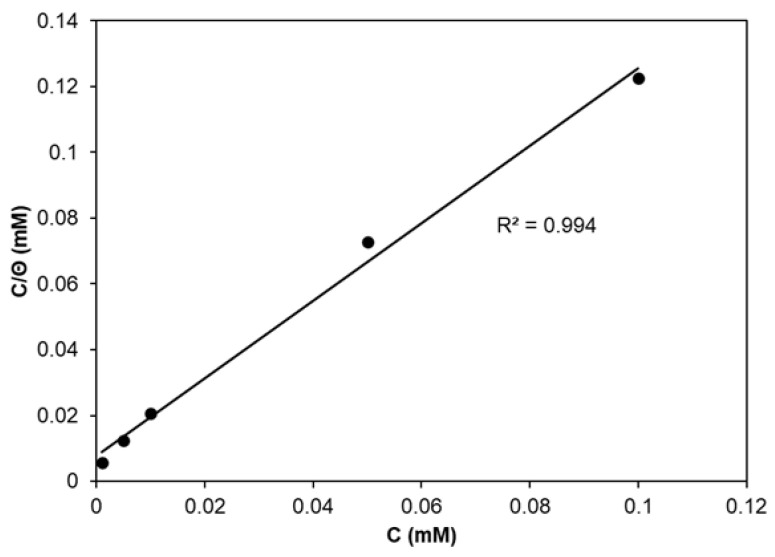
Langmuir isotherm of the adsorption CA on Zn surface in 0.1 M NaCl.

The results of PDP measurements can be used in fitting the suitable adsorption isotherm [51]. The best fitting for CA as the corrosion inhibitor was obtained for the Langmuir adsorption isotherm. The dependence of *C*/*Θ* vs. *C* (*C* − CA concentration) is presented in Figure 2 in the form of a straight line with the correlation coefficient value close to unity (R^2^ = 0.994). The slope of that straight line is 1.11 and suggests that the adsorbed molecules of CA form a monolayer on Zn surface without interactions between them. The intercept of the straight line presenting the adsorption isotherm is described by the following equation [2,3]:

(10) $$\frac{C}{\theta }=\frac{1}{K_{ads}}+C$$

It was used in calculations of the adsorption equilibrium constant (*K_ads_*). Its value is 153 L mM^−1^ and is related to the free energy of adsorption described by the equation [3,42]:

(11) $${∆G}_{ads}^{0}=-55.5RTlnK_{ads}$$

where *R* denotes the universal gas constant, *T* is the absolute temperature and 55.5 is the water solution concentration expressed in mol L^−1^ unit. The adsorption free energy calculated for CA adsorbed on Zn is 39.5 kJ mol^−1^. The negative sign of ${∆G}_{ads}^{0}$ denotes that the adsorption of CA proceeds spontaneously on Zn surface. Its value is related to the mixed physisorption–chemisorption [8,52].

**Figure 3:**
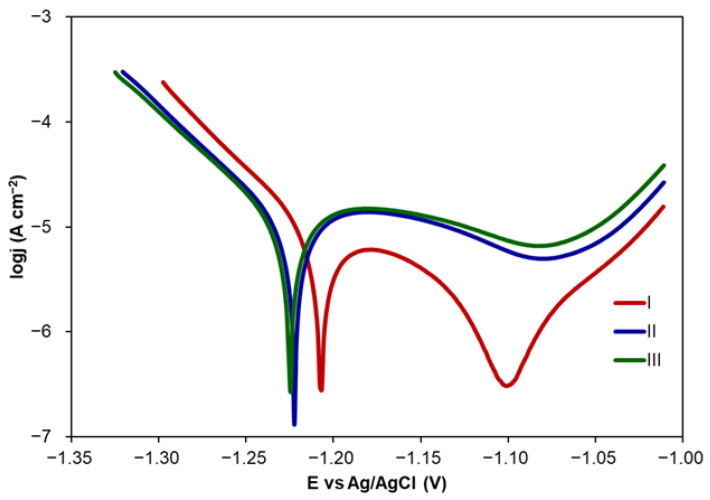
Tafel plots recorded for three consecutive measurements for Zn performed in 0.1 M NaCl with the addition of CA at the concentration of 0.1 mM.

The effect of a CA inhibitor on Zn corrosion vs. immersion time in NaCl was investigated in three consecutive PDP measurements performed at a CA concentration of 0.1 mM. The interval between two consecutive measurements was 70 min including OCP and PDP measurements’ duration. Tafel plots obtained from PDP curves are presented in Figure 3. The corrosion parameters calculated from Tafel plots are shown in Table 2.

The inhibition efficiency of CA determined in three consecutive measurements was calculated in relation to corrosion rates determined for Zn in 0.1 M NaCl, also in three consecutive measurements. The increasing immersion time resulted in a shift of E_corr_ towards more negative values. This indicates that Zn samples are more thermodynamically susceptible to the corrosion process. At the same time, the corrosion rate increased in the second measurement and was almost stable in the third measurement. The *IE* value decreased from 82 to about 70% indicating deterioration of CA inhibitive properties. However, its value was still relatively high. The Tafel plot shape recorded for the first measurement reveals the presence of the passive region at the potential of −1.1 V that significantly diminishes in the second and third measurements. Slightly visible passive regions are also observed for lower concentrations of CA (Figure 1).

**Figure 4:**
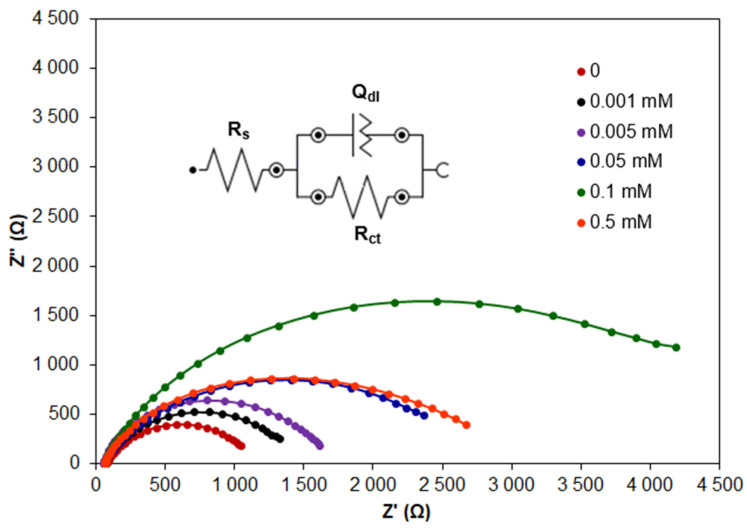
Exemplary Nyquist plots recorded for Zn samples in 0.1 M NaCl modified by the addition of CA at different concentrations.

In order to confirm the conclusions resulting from PDP measurements, EIS measurements were performed for Zn samples in NaCl with the addition of CA at concentrations in the range of 0.001–0.5 mM. Exemplary Nyquist plots are presented in Figure 4.

**Figure 5:**
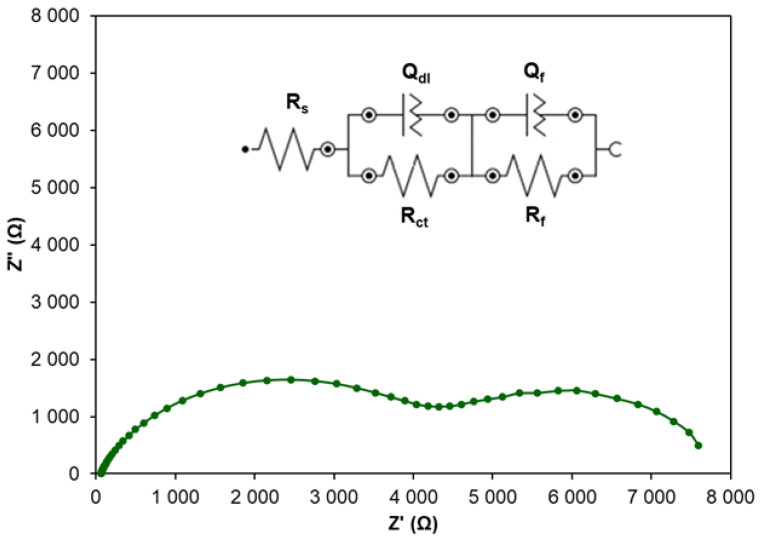
Nyquist plot recorded for Zn sample in 0.1 M NaCl modified by the addition of CA at the concentration of 0.1 mM.

Electrochemical impedance spectroscopy is a technique commonly applied in studies of electrical properties of metal/solution interfaces and is especially useful in investigating corrosion and adsorption phenomena. Nyquist plots recorded for Zn samples in NaCl with the addition of CA at various concentrations reveal a similar shape in the form of a semicircle with an increasing diameter. A larger diameter indicates higher resistance to corrosion. Only in the case of CA at the concentration of 0.1 mM is the shape of the Nyquist plot different, and two loops are clearly visible (Figure 5). The appearance of the second semicircle can be attributed to the formation of a dense and more protective layer of CA adsorbed on the Zn surface. This layer can be described by an additional (QR) equivalent electrical circuit.

In the high frequency region, the semicircle visible in all Nyquist plots has capacitive properties and is attributed to the charge transfer resistance of zinc in the absence and presence of the inhibitor. In the case of NaCl solution without the inhibitor, the diameter of the semicircle is small, while the addition of the inhibitor increases the diameter showing the increased resistance to corrosion. However, at the concentration of CA higher than 0.1 mM, the semicircle diameter decreases, and Nyquist plots are again in the form of a single semicircle. This proves that the CA concentration of 0.1 mM is optimal and highly effective in preventing the charge transfer reaction causing a significant decrease in the corrosion rate.

The obtained EIS data were fitted to the equivalent electrical circuit model using NOVA software ver. 2.1.7. In the case of all CA concentrations except for 0.1 mM, the equivalent circuit is attributed to the model [R_s_(Q_dl_R_ct_)] (Figure 4) and consists of the following elements: R_s_—solution resistance, *R_ct_*—charge transfer resistance and *Q_dl_*—constant phase element. The last element replaces the capacitance (*C_dl_*) of the electrical double layer in order to accommodate deviations from the ideal capacitive response [42], and its impedance (*Z*) is described by the equation [48]:

(12) $$Z_{\left(Q\right)}\left(\omega \right)=Q^{-1}\cdot {\left(j\omega \right)}^{-n}$$

This element is attributed to the heterogeneity of the Zn/NaCl interface.

In the case of 0.1 mM CA, the EIS data are described by the equivalent electrical circuit attributed to the model [R_s_(Q_dl_R_ct_)(Q_f_R_f_)] due to the presence of two semicircles (Figure 5) in the Nyquist plot. Q_f_ and *R_f_* elements are ascribed to the dense film of CA adsorbed on the Zn sample. The goodness of fit was evaluated based on the chi-squared parameter, whose values were in the range of 3.8 × 10^−3^–2.46 × 10^−2^. The resulting fitted EIS data are presented in Table 3 and are useful for evaluating the corrosion inhibition by CA.

The total polarisation resistance *R_p_* can be calculated using the following equation [3,48]:

(13) $$R_{p}=R_{ct}+R_{f}$$

and can be used in calculating inhibition efficiency (*IE*) according to the equation:

(14) $$IE\left(\%\right)=\frac{R_{p(inh)}-R_{p}}{R_{p(inh)}}\cdot 100$$

where *R*_*p*(*inh*)_ and *R_p_* is the polarisation resistance determined in the presence and in the absence of CA inhibitor, respectively.

It is obvious that the *R_p_* value is a measure of charge transfer across the Zn/solution interface and is inversely proportional to the corrosion rate. Thus, *R_p_* values recorded in the presence of CA are higher than in the absence of the inhibitor. At the same time, a decrease in double electrical layer capacitance (*C_dl_*) is observed. *C_dl_* was calculated according to the following equation [53,54]:

(15) $$C_{dl}=Q_{dl}(2\pi f_{\max}{)}^{n-1}$$

where *n* is a parameter describing surface irregularity, and *f_max_* denotes the frequency at which the imaginary part of impedance (Z″) has the highest value.

It was observed that an increasing CA concentration up to 0.1 mM lowers the value of *C_dl_* and increases the value of *R_p_*. This is due to the increase in thickness of the electrical double layer related to the adsorption of CA and to the decrease in the corrosion rate, respectively. The results of EIS measurements confirmed that the CA concentration of 0.1 mM is optimal.

#### 2.1.2. Corrosion Inhibition by CA Coatings

Considering the application of CA as a potential corrosion inhibitor for historical artefacts, CA coatings were formed on Zn samples. CA dissolved in ethanol at various concentrations was applied to Zn surface in different numbers of layers. Ethanol was used due to its high CA solubility in contrast to CA solubility in water. The concentration of CA was changed from 5 to 30 mM. Each layer was dried in a warm air stream in order to evaporate ethanol.

**Figure 6:**
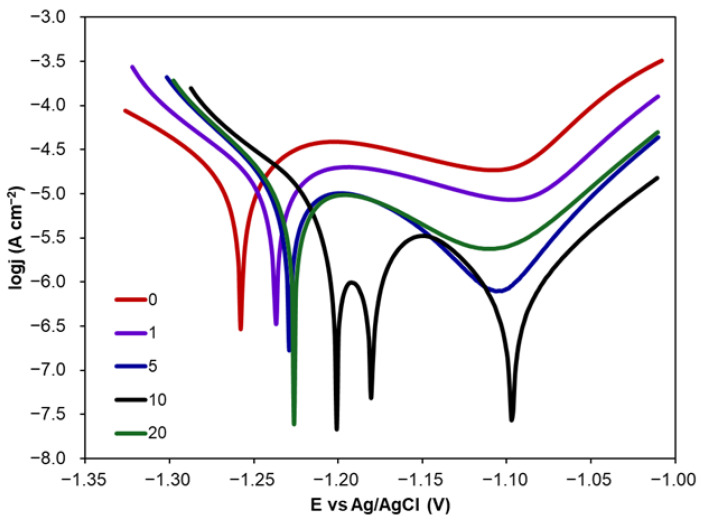
Tafel plots recorded for Zn coated with different numbers of layers (1, 5, 10 and 20) formed from 10 mM CA solution.

Similarly to the case of CA addition, PDP curves were recorded and transformed into Tafel plots. Exemplary Tafel plots recorded for Zn samples covered with various numbers of CA layers from 10 mM CA solution are presented in Figure 6. The corrosion parameters, E_corr_, *j_corr_*, b_a_ and b_c_ Tafel slopes, were determined from Tafel curves and used in the calculations of *CR* and *IE*. Their values are presented in Table 4.

Figure 6 shows that an increase in layer number results in the shift of E_corr_ towards more positive values in comparison with E_corr_ value (−1.258 V) determined for uncoated Zn in 0.1 M NaCl. This shift is not higher than 60 mV but indicates that CA coatings cause a slightly higher susceptibility of Zn to corrosion. Tafel slopes b_a_ and b_c_ are slightly lower than those determined for uncoated Zn in NaCl which proves that there is no change in corrosion mechanism. The decreasing values of *j_corr_* and consequently in *CR* values show that CA coatings decrease the corrosion rate. The higher the number of CA layers, the higher the *IE* values observed. However, *CR* values calculated for 10 and 20 layers were almost constant except for CA solutions with the concentrations of 5 and 10 mM. A comparison of *CR* and *IE* values allows one to conclude that the highest inhibitive effect of CA coating towards Zn corrosion is observed for 10 CA layers formed on Zn surface using CA solution at the concentration of 10 mM. Its inhibitive efficiency was 94.6%. In this case, a higher number of CA layers (20) resulted in a decrease in *IE* to 81.5%. Such an effect can be caused by the fact that CA coatings with a higher number of layers can be less uniform and dense, and therefore more susceptible to electrolyte penetration.

**Figure 7:**
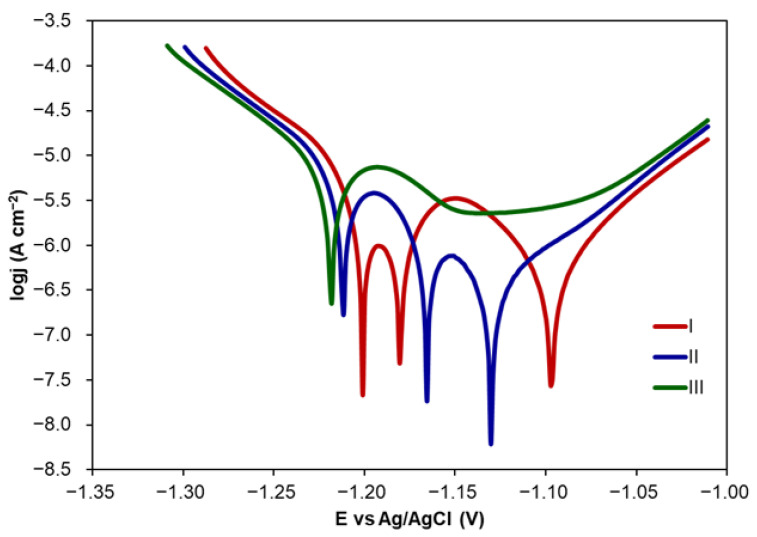
Tafel plots recorded for three consecutive measurements for Zn coated with 10 layers of CA (10 mM), performed in 0.1 M NaCl.

In order to check the stability of the coating with 10 layers of CA formed with the application of CA solution with the concentration of 10 mM, three consecutive PDP measurements were performed. Tafel plots obtained from PDP curves are presented in Figure 7. The corrosion parameters calculated from Tafel plots are shown in Table 5.

The comparison of E_corr_ shows a slight decrease in its value over the time of immersion in NaCl solution. This means that Zn samples with CA coating (10 layers) become a little bit more susceptible to corrosion phenomena. At the same time, *j_corr_* and *CR* values increase while *IE* values decrease. *IE* values were calculated in relation to *CR* determined for uncoated Zn samples also in three consecutive measurements. Even though *IE* becomes lower, its value in the third measurement is still relatively high. A decrease in *IE* values can be explained by the partial destruction of a coating and electrolyte penetration inside layers. Since CA coatings do not change the colour and appearance of Zn samples, they can be potentially used in corrosion protection of historical artefacts.

The Tafel plots presented in Figure 6 and Figure 7 reveal different shapes in the case of 10 layers of CA coating formed from CA solution at the concentration of 10 mM. There is one well-shaped active–passive peak at the potential of about −1.20 V. The second and third E_corr_ can be attributed to different corrosion products forming passive layers inhibiting corrosion and to localised (pitting) corrosion initiation, respectively [55,56]. Thus, cyclic PDP measurements were performed for uncoated Zn and Zn with CA coating including 10 layers (10 mM).

**Figure 8:**
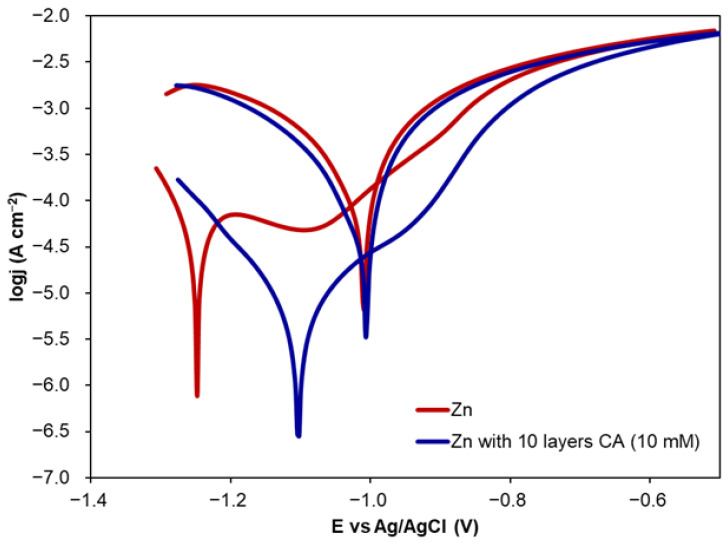
Cyclic PDP curves recorded for uncoated Zn and Zn coated with CA (10 layers, 10 mM).

In the case of both cyclic PDP curves (Figure 8), in the reverse scan, a small positive hysteresis is observed. This can be ascribed to the type of localised corrosion resulting in the oxidation of zinc [57]. Thus, it can be concluded that CA coating is not dense, and electrolyte can penetrate it causing the formation of pits.

**Figure 9:**
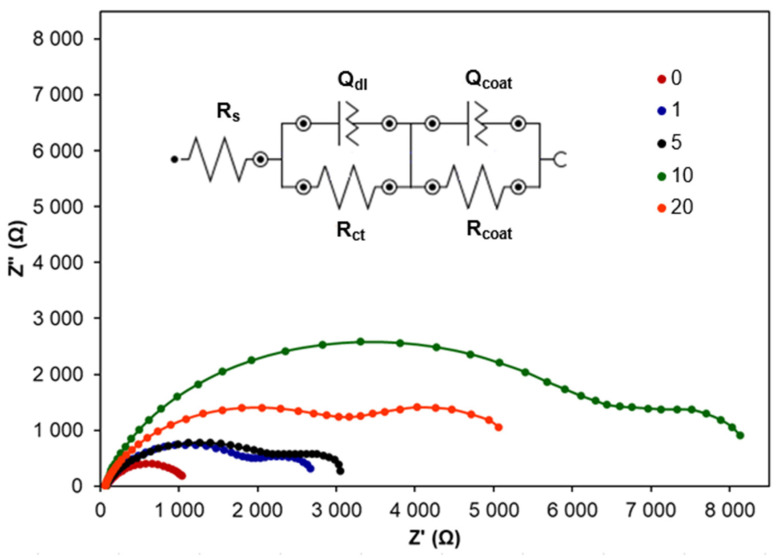
Nyquist plots for Zn samples coated with various numbers of CA layers (10 mM CA) recorded in 0.1 M NaCl.

In order to characterise the electrical properties of the interface between Zn with CA coatings and electrolyte solution, EIS measurements were performed. Nyquist plots recorded Zn coated with different numbers of layers are presented in Figure 9.

In the case of all Nyquist plots recorded for Zn with CA coatings, two semicircles can be distinguished. In the region of high frequencies, the semicircle is ascribed to the double electrical layer charge transfer reaction, while the semicircle at low frequencies is attributed to CA coatings. The equivalent electrical circuit presented in Figure 9 gave the best fit for the obtained data. This circuit attributed to the model [R_s_(Q_dl_R_ct_)(Q_coat_R_coat_)] includes Q_coat_ and R_coat_ elements ascribed to CA coatings on Zn surface. The resulting fitted EIS data presented in Table 6 were used for evaluating the corrosion inhibition by CA coatings with different numbers of layers. The goodness of fit was evaluated based on the chi-squared parameter, whose values were in the range of 1.24 × 10^−2^–2.06 × 10^−2^.

The increasing diameter of the first semicircle proves the increasing corrosion resistance of Zn with CA coatings with the number of layers increasing up to 10. Although R_coat_ determined for 20 layers is higher than for 10 layers, the total polarisation resistance *R_p_* is clearly higher for CA coating with 10 layers. This means that 10 layers exhibit better anticorrosion properties. In the case of 20 layers, the coating is probably not uniform and dense. These results support the conclusions resulting from PDP measurements. At the same time, the capacitance of the electrical double layer decreases with the increasing number of CA layers, and its lowest value occurs for 10 layers. This number of CA layers increases IE significantly and seems to be optimal for Zn protection against corrosion in NaCl.

### 2.2. Morphological Characterisation of Zn Surface

The surface morphology of Zn samples with and without CA coatings before and after immersion in NaCl was first studied by using a scanning electron microscope (SEM) coupled with EDS.

**Figure 10:**
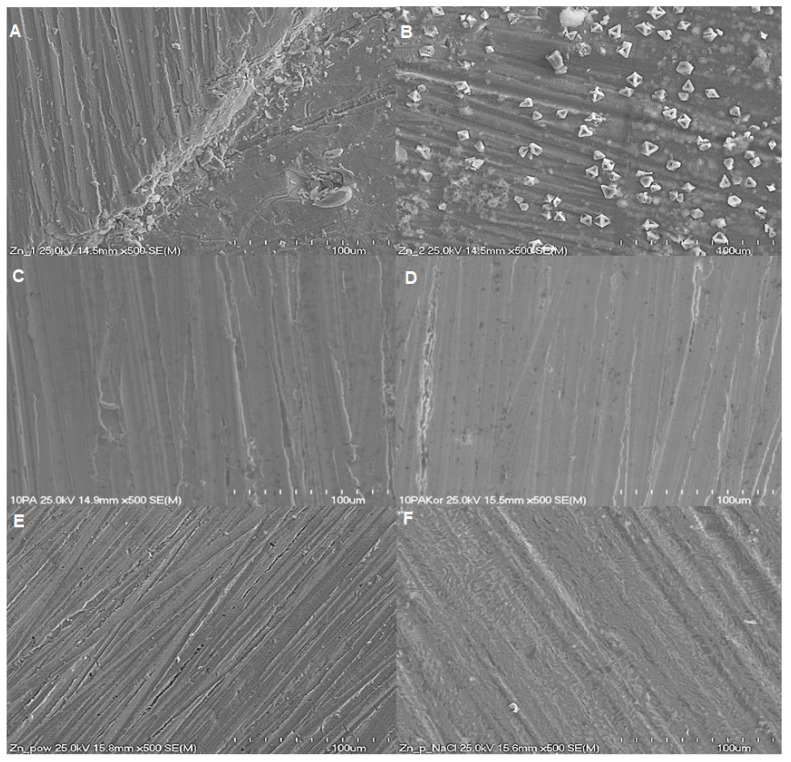
SEM images of pure Zn (**A**), Zn after exposition to 0.1 M NaCl (**B**), Zn with 10 protective layers formed from 10 mM CA solution (**C**), Zn with 10 protective layers after the exposition to 0.1 M NaCl (**D**), Zn with 20 protective layers formed from 10 mM CA solution (**E**) and Zn with 20 protective layers after the exposition to 0.1 M NaCl (**F**).

**Figure 11:**
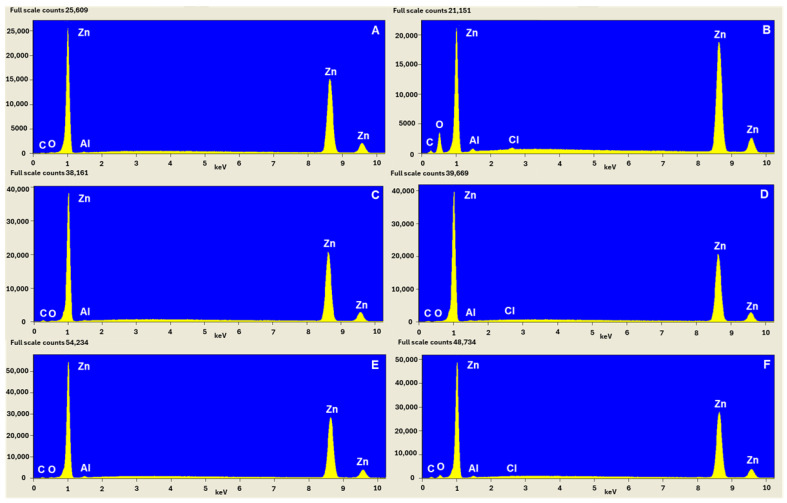
EDS spectra recorded for pure Zn sample (**A**), Zn after corrosion in NaCl (**B**), Zn sample with 10 layers of caffeic acid before (**C**) and after corrosion in NaCl (**D**), Zn sample with 20 layers of caffeic acid before (**E**) and after corrosion in NaCl (**F**).

The pure Zn samples were characterised by some scratches originating from the method of preparing samples for the measurements. After the exposition to the corrosive medium (0.1 M NaCl), Zn sample surface showed the presence of corrosion products in the form of crystals (Figure 10B). The EDS spectra recorded for the pure Zn sample and Zn sample after exposition to NaCl solution (Figure 11A,B) confirm the formation of commonly detected corrosion products containing ZnO, Zn(OH)_2_ and Zn_5_(OH)_8_Cl_2_⋅H_2_O according to the reactions (3) to (8).

The spectrum of EDS consists mainly of C, O and Zn. The basic composition of Zn surface is Zn (81%) with a small content of O and C introduced from the atmosphere. The sample after exposition to NaCl has a different composition. The content of O increased to 30%, while the content of Zn decreased to 49% with simultaneous appearance of Cl. This confirms the formation of the above-mentioned corrosion products.

Figure 10C,E present the surface of the Zn sample with protective coatings, containing 10 and 20 layers, respectively, formed from CA solution. The surface of the Zn sample with 10 protective layers is clearly more uniform with fewer visible scratches (Figure 10C) in comparison to Zn with 20 layers and pure Zn. After the immersion in NaCl solution, almost no corrosion products can be observed in the case of Zn samples with 10 layers (Figure 10D). Taking into consideration the O content, it can be concluded that only small amounts of ZnO and Zn(OH)_2_ were formed on Zn with 20 layers (Table 7). Furthermore, the surface of the Zn sample is less uniform and compact in the case of 20 layers in comparison to 10 layers both before and after immersion in NaCl. SEM images proved that the better corrosion inhibition observed for Zn samples modified with CA layers can be attributed to the deposited protective layers. However, better results were obtained for 10 protective layers. The spectra of EDS recorded for Zn sample with 10 and 20 layers of CA are very similar to the spectrum of the pure Zn sample (Figure 11). However, the content of C is higher, probably due to the carbon atoms constituting caffeic acid layers on Zn surface. The Zn sample modified with CA and after corrosion in NaCl shows three times lower content (about 10%) and significantly lower content of O for 20 and 10 layers of CA, respectively. The content of Cl is very low in the case of three Zn samples after immersion in 0.1 M NaCl, but its lowest value is observed for Zn covered with 10 layers of CA. This confirms that the modification of Zn surface with CA results in the formation of much lower amounts of corrosion products due to inhibition of corrosion by CA coatings.

**Figure 12:**
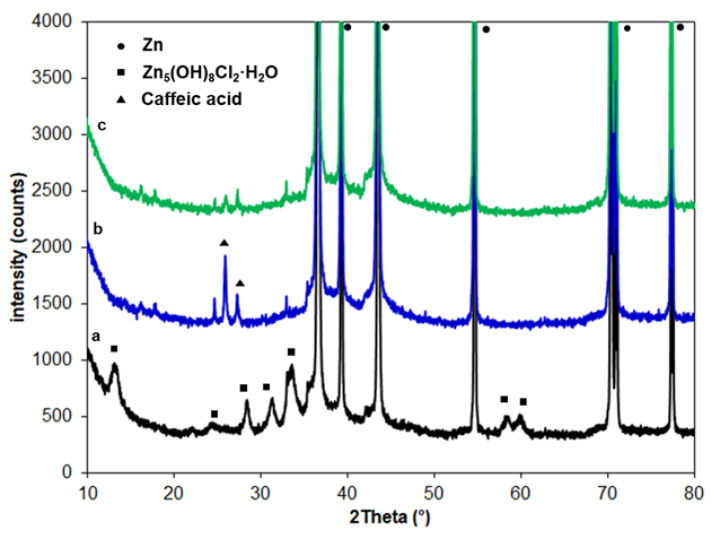
Zoomed XRD patterns of Zn after corrosion in 0.1 M NaCl (a), Zn coated with CA layers (b) and Zn coated with CA layers after corrosion in 0.1 M NaCl (c).

The phase composition of the studied samples was characterised using X-ray diffraction (XRD). The XRD patterns are presented in Figure 12. All patterns in Figure 12 reveal the presence of a crystalline hexagonal structure of the Zn phase with diffraction peaks corresponding to the (002), (100), (101), (102), (103) and (110) crystal planes, located at 2θ positions of 36.3°, 38.0°, 43.2°, 54.3°, 70.1° and 70.7°, respectively. The Zn phase was confirmed via the JCPDS PDF-2 card no. 00-004-0831. Figure 12 (curve a) presents the XRD pattern of Zn after corrosion in 0.1 M NaCl. In addition to the dominant peaks associated with the Zn phase, several peaks of relatively low intensity were also observed, corresponding to the Zn_5_(OH)_8_Cl_2_·H_2_O phase (JCPDS PDF-2 card no. 01-088-88940). These findings are consistent with those reported by Meng et al. [45]. Figure 12 (curve b) shows the diffraction pattern of Zn coated with CA layers. Small peaks appear at 2θ angles of approximately 25.9° and 27.0°. Based on the single-crystal data [58], a polycrystalline standard was generated. These maxima correspond to caffeic acid. The diffraction patterns of the Zn sample coated with CA layers of caffeic acid and subjected to corrosion in 0.1 M NaCl (Figure 12, curve c) show no peaks resulting from corrosion products. Additionally, all diffraction patterns feature a peak at a 2θ angle of approximately 33.0°, which could not be assigned to a specific phase, likely due to the absence of a corresponding reference pattern.

The conclusions resulting from XRD measurements are confirmed by data obtained using the time-of-flight secondary ion mass spectrometry (TOF-SIMS) technique. TOF-SIMS was used to study the corrosion products on the Zn samples exposed to NaCl solution.

TOF-SIMS spectra were recorded for the tested samples of Zn and Zn covered with CA layers (ethanolic solution) after immersion in 0.1 M NaCl. The emission of ZnO_2_^−^ and ZnO_2_H^−^ ion at *m*/*z* 96 and 97 was selected as indicative of zinc oxidation products, ZnO and Zn(OH)_2_, on the sample surface. In addition, a peak at *m*/*z* 115 can be identified as a fragment ion ZnOCl^−^ ascribed to Zn_5_(OH)_8_Cl_2_⋅H_2_O as the corrosion product.

**Figure 13:**
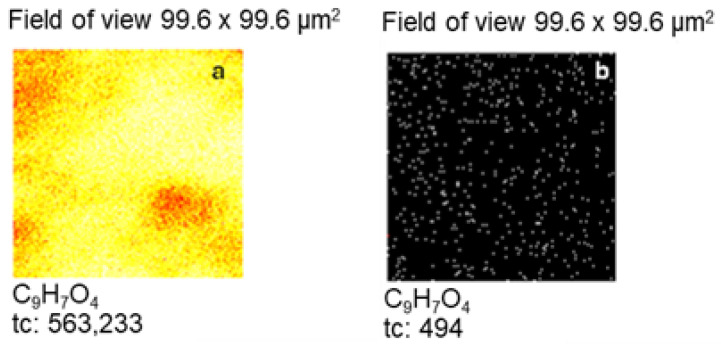
TOF-SIMS images of the Zn sample surface covered with CA layers (**a**) and the surface of the zinc sample below CA layers (**b**).

In order to investigate the protective properties of CA, the intensities of ZnO_2_^−^, ZnO_2_H^−^ and ZnOCl^−^ signals from the Zn sample below CA layers were analysed. Measurements were performed using ion sputtering of CA layers followed by TOF-SIMS analysis of the uncovered zinc surface. The uniform layer of CA on the Zn sample (after immersion in NaCl) before ion sputtering is confirmed by the TOF-SIMS image in Figure 13a, whereas Figure 13b indicates successful removal of CA layers via ion sputtering before TOF-SIMS analysis of the sample surface below the CA layers.

**Figure 14:**
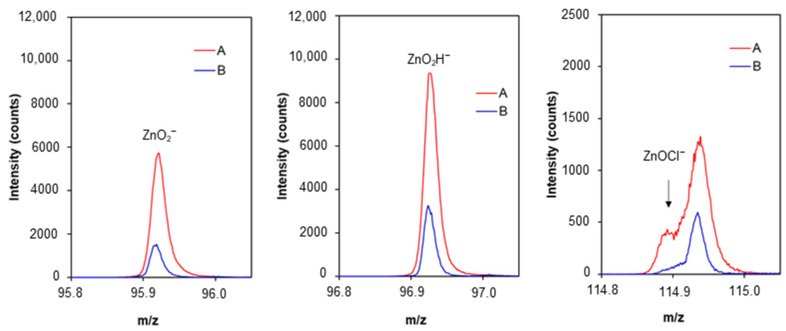
TOF-SIMS spectra of the subsurface layers of the bared pure Zn sample (A) and CA protected sample (B).

**Figure 15:**
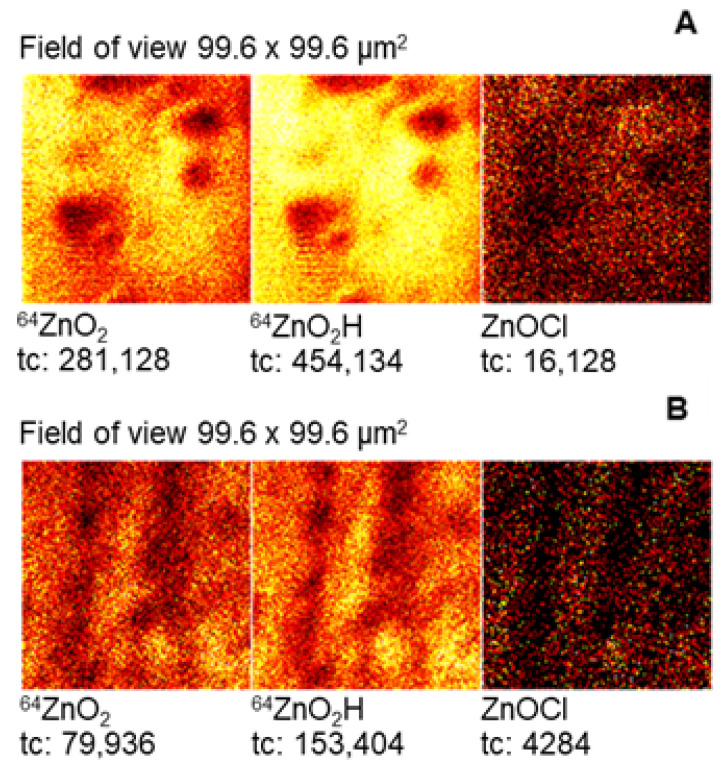
TOF-SIMS images of the subsurface layers of the bared pure Zn sample (**A**) and CA protected sample (**B**).

For comparison purposes, the ion sputtering for the same time as for CA-protected zinc was applied to the Zn sample without CA protective layers. The corresponding TOF-SIMS spectra and ion images recorded for samples after immersion in NaCl are presented in Figure 14 and Figure 15, respectively.

The TOF-SIMS spectra and images clearly show that the intensity of the ions characteristic for corrosion products is much higher for the sample without CA layers. This fact confirms that CA layers on the surface of Zn samples exhibit protective properties against corrosion in NaCl. In addition, TOF-SIMS images show that corrosion products on the bared pure Zn samples are unevenly distributed, indicating the presence of pitting corrosion.

## 3. Materials and Methods

### 3.1. Materials Preparation

In this study, pure Zn (99.9%) samples were used. All samples had exposed surface of 1 cm^2^ and 1 mm thickness. Before the measurements, each sample was first grinded with K180 sandpaper (LUX Tools, Wermelskirchen, Germany) and then degreased with acetone (99%, Chempur, Piekary Slaskie, Poland).

**Figure 16:**
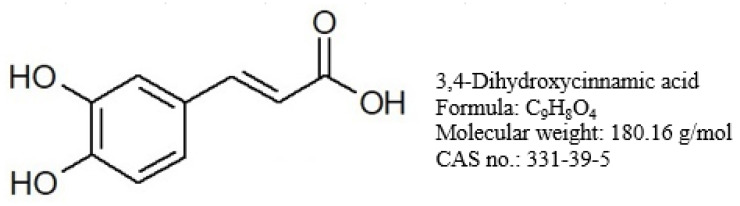
The structure of caffeic acid.

0.1 M NaCl solution was prepared by dissolving NaCl (99.9%, Chempur, Piekary Slaskie, Poland) powder in doubly distilled water in a 2000 mL volumetric flask. Caffeic acid solutions in NaCl were prepared by dissolving the appropriate mass of caffeic acid (Figure 16) powder (98%, Sigma-Aldrich, Saint Louis, MO, USA) in 1 mL of ethanol (99.9%, Supelco, Bellefonte, PA, USA) and then quantitively transferring to a 200 mL volumetric flask and filling with NaCl.

Caffeic acid solutions for sample coatings were formed on Zn surface by brushing samples with the acid dissolved in ethanol (5, 10, 20 and 30 mM). After the formation of each layer, the samples were dried in warm air.

### 3.2. Electrochemical Measurements

A potentiodynamic polarisation (PDP) method was used to determine the corrosion parameters of the tested samples. Measurements were taken using Autolab PGSTAT 302N potentiostat (Metrohm Autolab B.V., Utrecht, The Netherlands) in a four-electrode system. The silver chloride electrode (Ag/AgCl) was used as a reference electrode, two steel rods were used as auxiliary electrodes, and the zinc plates acted as working electrodes. All experiments were conducted at room temperature. Before each PDP measurement, a 1 h open circuit potential (OCP) measurement was taken to achieve a steady-state open circuit potential. Next, samples were polarised in the range of OCP ± 0.3 V with a scan rate of 1 mV/s. PDP curves (j = f(E)) recorded in tested solutions were converted to Tafel plots (logj = f(E)) using NOVA 2.1.7 software. The intersection between the anodic and the cathodic Tafel slopes defines the corrosion current density (*j_corr_*) and corrosion potential (E_corr_), and cathodic (b_c_) and anodic (b_a_) Tafel slopes.

The corrosion rate (*CR*) in mm year^−1^ was calculated using the Stern–Geary equation through Faraday’s law [59]:

(16) $$CR=\frac{j_{corr}\cdot K\cdot EW}{\rho }$$

where *K*—constant equals to 3270 µm (A·cm·year)^−1^, *EW*—equivalent weight of metal (g mol^−1^) and *ρ*—metal density (g cm^−3^). The inhibition efficiency (*IE*) of the caffeic acid was calculated according to the following equation:

(17) $$IE\left(\%\right)=\frac{j_{corr}-j_{corr\left(inh\right)}}{j_{corr}}\cdot 100$$

where *j*_*corr*(*inh*)_ denotes the current density determined in the presence of CA.

EIS experiments were performed at the OCP value by applying a sinusoidal signal with an amplitude of 10 mV and in the frequency range of 0.001 to 100,000 Hz. The results of EIS measurements are presented in the form of Nyquist plots and are useful in determining both the corrosion and inhibition mechanism as well as the kinetics of the corrosion process. The data obtained from PDP and EIS measurements were collected and analysed using NOVA ver. 2.1.7 software dedicated to the Autolab PGSTAT 302N.

### 3.3. Surface Characterisation of Zn Samples

The changes in morphology of the tested Zn samples were determined using SEM-EDS, XRD and TOF-SIMS techniques.

The surface morphology and chemistry of Zn samples with and without CA coatings were determined via scanning electron microscopy SEM (S-4700, Hitachi, Tokyo, Japan) coupled with energy dispersive X-ray analysis EDS (Noran System, Thermo Fisher Scientific, Waltham, MA, USA). Images were recorded at ×500 magnification, scale bar 100 μm. The applied voltage was 25 kV in each measurement.

The room-temperature powder X-ray diffraction pattern was collected using a PANalytical X’Pert Pro MPD diffractometer in Bragg–Brentano reflection geometry. Copper CuKα radiation was used from a sealed tube. Data were collected in the 2θ range of 4–80° with a step size of 0.0167° and an exposure time of 30 s per step. A PANalytical X’Celerator detector, based on Real-Time Multiple Strip technology and capable of simultaneously measuring intensities over a 2θ range of 2.122°, was used.

The secondary ions mass spectra were recorded using a TOF-SIMS IV mass spectrometer (IONTOF GmbH, Muenster, Germany). The instrument is equipped with a Bi liquid metal ion gun and high mass resolution time of flight mass analyser. Secondary ion mass spectra were recorded for an approximately 100 × 100 μm^2^ area of the sample surface. During spectrum acquisition, the analysed area of the sample surface was irradiated with pulses of 25 keV Bi^3+^ ions at a 10 kHz repetition rate. The average current of the primary ion pulsed beam was 0.2 pA, and the pulse duration was 1 ns. The time of spectrum acquisition was 30 s giving an ion dose below a static limit of 1 × 10^13^ ions cm^−2^. Ion sputtering with 3 keV Cs^+^ ions for 50 s was used to remove CA layers in order to perform the TOF-SIMS analysis of chemical composition of the sample below CA layers after corrosion in NaCl solution. Similarly, the spectra of the samples without CA layers were recorded from the sample surface cleaned by sputtering with 3 keV Cs^+^ ions for 50 s prior to the measurements. The current of the Cs^+^ beam was 13 nA and the sputtered area was 150 × 150 μm^2^. A pulsed 20 eV electron flood gun was used for charge neutralisation during spectra acquisition.

## 4. Conclusions

The effect of caffeic acid as a green corrosion inhibitor for Zn was investigated in 0.1 M NaCl. The corrosion parameters were determined using potentiodynamic polarisation and electrochemical impedance spectroscopy methods. The results of the experiments clearly show that the highest inhibition efficiency (82%) was achieved for a CA concentration of 0.1 mM. CA appears to be a mixed-type corrosion inhibitor with more control of anodic dissolution of Zn. Its inhibition effect is related to the adsorption on Zn surface with formation of the monolayer, which was proved by fitting the obtained data to the Langmuir isotherm. The higher concentrations of CA resulted in lower inhibition efficiency probably due to the worsening of the adsorption of CA molecules on the Zn surface. The obtained results were confirmed via EIS measurements. A similar *IE* value (86%) was also achieved for 0.1 mM CA, for which the capacitance of the electrical double layer was the lowest. The increase in the immersion time in NaCl resulted in a slight deterioration of the inhibition efficiency of CA.

The influence of CA on the corrosion rate of Zn samples was also investigated via CA application in the form of protective coatings. The results obtained from PDP indicate that the highest *IE* value (95%) was achieved for ten layers of CA obtained from ethanol solution of CA (10 mM). The *IE* value was even higher than in the case of CA addition to NaCl. Moreover, the increasing immersion time in NaCl resulted in slightly lower *IE* values but higher than in the case of CA addition to NaCl. The obtained results were confirmed via EIS measurements. The investigation of changes in the morphology of Zn samples proved the presence of CA coatings on the Zn surface and typical Zn corrosion products in NaCl. However, in the case of CA coatings, a noticeably lower amount of corrosion products was determined on Zn surface.

The application of CA in the form of protective coatings seems to be an effective method of Zn protection. Additionally, CA coatings do not change the colour and appearance of the protected samples. Thus, this method can potentially be applied in corrosion protection of historical heritage artefacts made of brass since Zn is one of its components.

## Figures and Tables

**Table 1:** The corrosion parameters obtained from Tafel curves recorded for Zn in 0.1 M NaCl modified by the addition of CA at different concentrations.

CA Conc. (mM)	E_corr_ (V)	b_a_ (V dec^−1^)	b_c_ (V dec^−1^)	*j_corr_* (A cm^−2^)	*CR* (mm year^−1^)	*IE* (%)	*Θ* (−)
0	−1.258	0.069	−0.067	1.09 × 10^−5^	0.1634	−	−
0.001	−1.249	0.040	−0.039	6.79 × 10^−6^	0.1018	37.7	0.38
0.005	−1.256	0.041	−0.047	6.48 × 10^−6^	0.0971	40.6	0.41
0.01	−1.243	0.042	−0.039	5.64 × 10^−6^	0.0845	48.3	0.48
0.05	−1.227	0.034	−0.024	3.40 × 10^−6^	0.0509	68.8	0.69
0.1	−1.207	0.035	−0.020	2.00 × 10^−6^	0.0301	81.6	0.82
0.5	−1.164	0.069	−0.058	2.96 × 10^−6^	0.0444	72.8	0.73

**Table 2:** Corrosion parameters determined for three consecutive measurements for Zn, performed in 0.1 M NaCl with the addition of 0.1 mM CA solution.

Measur. No.	E_corr_ (V)	b_a_ (V dec^−1^)	b_c_ (V dec^−1^)	*j_corr_* (A cm^−2^)	*CR* (mm year^−1^)	*IE* (%)
0.1 mM NaCl						
1	−1.258	0.069	−0.067	1.09 × 10^−5^	0.1634	−
2	−1.250	0.103	−0.072	1.00 × 10^−5^	0.1502	−
3	−1.244	0.145	−0.079	9.96 × 10^−6^	0.1493	−
0.1 mM NaCl + 0.1 mM CA						
1	−1.207	0.030	−0.020	2.00 × 10^−6^	0.0301	81.6
2	−1.222	0.036	−0.025	3.24 × 10^−6^	0.0486	67.6
3	−1.225	0.030	−0.023	3.00 × 10^−6^	0.0450	69.9

**Table 3:** Electrochemical impedance parameters of Zn samples in 0.1 M NaCl with and without the addition of CA inhibitor.

CA (mM)	R_s_ (Ω)	*R_ct_* (Ω)	*Q_dl_* (S·s)^n^	*n*	*R_f_* (Ω)	Q_f_ (S·s)^n^	n_f_	*IE* (%)	*C_dl_* (μF)
0	75.3	1093.2	3.375 × 10^−5^	0.779	-	-	-	-	13.52
0.001	64.3	1422.5	2.610 × 10^−5^	0.782	-	-	-	23.1	11.13
0.005	63.2	1478.9	3.247 × 10^−5^	0.847	-	-	-	26.1	11.29
0.01	63.3	1580.8	4.314 × 10^−5^	0.864	-	-	-	30.8	11.84
0.05	57.0	2560.4	1.921 × 10^−5^	0.744	-	-	-	57.3	7.06
0.1	62.2	4071.0	1.758 × 10^−5^	0.812	3807.9	5.926 × 10^−4^	0.771	86.1	6.23
0.5	62.9	2586.6	3.323 × 10^−5^	0.784	-	-	-	57.7	8.26

**Table 4:** The corrosion parameters obtained from Tafel curves recorded for Zn coated with different numbers of layers formed from CA solution of different concentrations.

Number of Layers	E_corr_ (V)	b_a_ (V dec^−1^)	b_c_ (V dec^−1^)	*j_corr_* (A cm^−2^)	*CR* (mm year^−1^)	*IE* (%)
5 mM CA						
1	−1.233	0.048	−0.037	4.69 × 10^−6^	0.0703	57.0
5	−1.232	0.044	−0.029	4.07 × 10^−6^	0.0610	62.7
10	−1.234	0.030	−0.025	3.52 × 10^−6^	0.0527	67.7
20	−1.224	0.038	−0.024	2.67 × 10^−6^	0.0400	75.5
10 mM CA						
1	−1.237	0.042	−0.034	5.26 × 10^−6^	0.0789	51.7
5	−1.229	0.025	−0.020	2.46 × 10^−6^	0.0369	77.4
10	−1.201	0.035	−0.012	5.95 × 10^−7^	0.0089	94.6
20	−1.226	0.021	−0.017	2.02 × 10^−6^	0.0303	81.5
20 mM CA						
1	−1.224	0.040	−0.023	2.72 × 10^−6^	0.0408	75.0
5	−1.221	0.037	−0.021	2.20 × 10^−6^	0.0330	79.8
10	−1.219	0.035	−0.021	2.12 × 10^−6^	0.0318	80.5
20	−1.222	0.031	−0.020	2.11 × 10^−6^	0.0317	80.6
30 mM CA						
1	−1.228	0.068	−0.039	5.65 × 10^−6^	0.0847	48.2
5	−1.223	0.058	−0.032	3.90 × 10^−6^	0.0584	64.3
10	−1.218	0.044	−0.022	2.12 × 10^−6^	0.0317	80.6
20	−1.219	0.034	−0.019	1.89 × 10^−6^	0.0283	82.7

**Table 5:** Corrosion parameters determined for three consecutive measurements for Zn coated with 10 layers of CA (10 mM), performed in 0.1 M NaCl.

Measur. No.	E_corr_ (V)	b_a_ (V dec^−1^)	b_c_ (V dec^−1^)	*j_corr_* (A cm^−2^)	*CR* (mm year^−1^)	*IE* (%)
1	−1.201	0.035	−0.012	5.95 × 10^−7^	0.0089	94.6
2	−1.212	0.041	−0.021	1.80 × 10^−6^	0.0270	82.0
3	−1.218	0.020	−0.023	2.11 × 10^−6^	0.0316	78.8

**Table 6:** Electrochemical impedance parameters of Zn samples with CA coatings recorded in 0.1 M NaCl.

Number of Layers	R_s_ (Ω)	*R_ct_* (Ω)	*Q_dl_* (S·s)^n^	*n*	R_coat_ (Ω)	Q_coat_ (S·s)^n^	n_coat_	*IE* (%)	*C_dl_* (μF)
0	75.3	1093.2	3.375 × 10^−5^	0.779	-	-	-	-	13.52
1	67.9	1974.3	2.864 × 10^−5^	0.777	942.8	1.292 × 10^−3^	0.868	62.5	13.27
5	63.9	1983.5	3.348 × 10^−5^	0.769	1417.9	9.707 × 10^−4^	0.696	67.9	13.42
10	69.0	5997.9	1.389 × 10^−5^	0.864	2921.7	6.387 × 10^−4^	0.789	87.7	8.97
20	62.5	3088.3	1.626 × 10^−5^	0.832	3035.7	1.071 × 10^−4^	0.797	82.1	10.63

**Table 7:** Elemental composition of pure Zn and Zn with 10 and 20 protective layers of CA, before and after immersion in 0.1 M NaCl.

Element	wt. (%)	
	Zn	Zn After Immersion
C	15.56	19.41
O	2.37	29.95
Al	1.13	1.85
Cl	0.00	0.28
Zn	80.94	48.51
	Zn 10 layers	Zn 10 layers after immersion
C	26.00	15.11
O	3.02	1.47
Al	3.25	3.33
Cl	0.00	0.02
Zn	67.73	80.07
	Zn 20 layers	Zn 20 layers after immersion
C	20.22	21.26
O	2.43	10.38
Al	1.87	1.93
Cl	0.00	0.11
Zn	75.48	66.32

## Data Availability

The data is contained within the article.
